# An Improvement of SPME-Based Sampling Technique to Collect Volatile Organic Compounds from *Quercus ilex* at the Environmental Level

**DOI:** 10.3390/metabo11060388

**Published:** 2021-06-14

**Authors:** Dalila Pasquini, Antonella Gori, Francesco Ferrini, Cecilia Brunetti

**Affiliations:** 1 Department of Agriculture, Food, Environment and Forestry (DAGRI), University of Florence, 50019 Sesto Fiorentino, Italy; antonella.gori@unifi.it (A.G.); francesco.ferrini@unifi.it (F.F.); cecilia.brunetti@ipsp.cnr.it (C.B.); 2 National Research Council of Italy, Institute for Sustainable Plant Protection (IPSP), 50019 Sesto Fiorentino, Italy; 3 VALUE Laboratory on Green, Health & Wellbeing, University of Florence and the Italian Horticultural Society, 50019 Sesto Fiorentino, Italy

**Keywords:** BVOCs, GC-MS, monoterpenes, *Quercus ilex*, SPME

## Abstract

Biogenic Volatile Organic Compounds (BVOCs) include many chemical compounds emitted by plants into the atmosphere. These compounds have a great effect on biosphere–atmosphere interactions and may affect the concentration of atmospheric pollutants, with further consequences on human health and forest ecosystems. Novel methods to measure and determine BVOCs in the atmosphere are of compelling importance considering the ongoing climate changes. In this study, we developed a fast and easy-to-handle analytical methodology to sample these compounds in field experiments using solid-phase microextraction (SPME) fibers at the atmospheric level. An improvement of BVOCs adsorption from SPME fibers was obtained by coupling the fibers with fans to create a dynamic sampling system. This innovative technique was tested sampling *Q. ilex* BVOCs in field conditions in comparison with the conventional static SPME sampling technique. The results showed a great potential of this dynamic sampling system to collect BVOCs at the atmosphere level, improving the efficiency and sensitivity of SPME fibers. Indeed, our novel device was able to reduce the sampling time, increase the amount of BVOCs collected through the fibers and add information regarding the emissions of these compounds at the environmental level.

## 1. Introduction

Trees evolved in arid and semi-arid environments, such as the Mediterranean basin, have developed several defense mechanisms to cope with drought, changing their physiology and metabolism. One of these mechanisms is the biosynthesis of Biogenic Volatile Organic Compounds (BVOCs), a large group of secondary metabolites, among which volatile isoprenoids (mono-, sesqui- and homo-terpenes) are the most important [1,2]. These compounds show noticeable functions in protection, defense and communication among plants, as well as between plants and other organisms. Their emissions are largely controlled by genetic and environmental conditions [3,4].

Most of the Mediterranean forest plants have been described as high BVOC emitters, in particular of monoterpenes, investing a great proportion of fresh assimilated carbon in their biosynthesis during the summer season [5,6,7,8]. Several studies have reported that BVOC emission is enhanced under abiotic stresses, such as drought and heat [9,10]. The estimated amount of isoprene and monoterpenes emitted by Mediterranean forests is about 4.5 kg km^−2^ day^−1^ [11], and it has a great impact on the atmospheric chemistry of this vulnerable habitat [11,12]. These compounds have a great effect on biosphere–atmosphere interactions by altering aerosol growth processes, cloud formation and, in general, modifying atmospheric reactivity [13]. Indeed, BVOCs oxidation, especially of monoterpenes, plays an important role in the atmospheric chemistry as precursors of tropospheric ozone (O_3_) and secondary organic aerosol (SOA) [14,15,16,17,18]. In particular, ozone is a greenhouse gas with detrimental effects on plant and human health [19,20]. In addition, monoterpenes have been shown to interact with human health in different ways, from stress relief to influencing immune function. In fact, recent studies have shown meaningful effects of BVOCs inhalation on the relief of stress-related mood disorders [21,22,23]. Terpenes, in addition to being largely utilized in the pharmaceutical industry, have recently been identified as the main chemical compounds responsible for the beneficial effects of the “forest bathing” therapy, an emerging popular practice consisting of immersing oneself in nature by mindfully using all five senses [24,25]. Increasing global concerns about the effects of atmospheric pollutants on human health and forest functioning are leading researchers to look for novel methods to collect and measure BVOCs in the atmosphere [26].

Up to date, BVOCs emission have been mainly investigated using plant and leaf enclosures connected to adsorption tubes or fibers followed by Gas Chromatography – Mass Spectrometry (GC−MS) analysis [27]. In particular, Solid-Phase MicroExtraction (SPME) fibers is a solvent free sampling technique and possesses useful characteristics to collect BVOCs in field conditions, since it is easy-to-handle, durable and provides repeatable results [28,29,30]. SPME can be used both in static (Static-HeadSpace—S-HS) and dynamic (Dynamic HeadSpace—D-HS) sampling enclosures of leaves or branches [31]. Between these two sampling systems, the D-HS has been recognized to provide more accurate measurements [32]. Indeed, measuring plants placed in an enclosed system, as in the case of S-HS, can result in unrealistically physiological conditions affecting gas exchanges and potentially altering BVOC emission rates of the plants [31,33]. SPME fibers can be also directly exposed to open air, a method largely applied to monitor and quantify volatile organic pollutants in urban environment through static sampling [34,35]. Moreover, SPME fibers have also been utilized for dynamic sampling at canopy level, by coupling with a fan-sampler [36] and, more recently, with a drone system [37]).

Several SPME sorbent coatings have been developed, such as divinylbenzene/polydimethylsiloxane (DVB/PDMS), which is used especially for the adsorption of semi-volatile analytes and larger volatile compounds, carboxen/polydimethylsiloxane (CAR/PDMS), suitable for small volatile molecules, and DVB/CAR/PDMS, which is indicated for the adsorption of an extended molecular weight range of analytes [30]. For this reason, in our study, we utilized the DVB/CAR/PDMS coating. In this study, DVB/CAR/PDMS fibers have been included in a dynamic sampling system characterized by a controlled and constant air flux, which allowed the adsorption of BVOCs on the fibers and the ability to reach the equilibrium retaining analytes with different molecular weights uniformly [38]. Our Dynamic BVOC Sampling System (DBSS), based on a previous study [36], was improved in the material used for the instrument to make it cheaper and easier to assemble. Our choice was to use aluminum instead of polyacetal plastic. Using this metal, all the components are easily available in any hardware store, and no glue was used for its assembly to avoid possible interferences in BVOCs analysis. The choice of aluminum was guided by the need to have a light and resistant instrument for field campaigns. Furthermore, the DBSS shape and its low weight allow it to be easily set up on a metal picket in the forest, while the metal net in front of the chamber provides a physical barrier to insects or debris, additionally creating a swirling flow. The DBSS was positioned at a height of 45 cm from the ground, which is optimal to limit wind action and manage the sampling operations [39]. Finally, the sampling duration of 4 h was chosen to obtain the best compromise between a long exposition time of SPME fibers [40] and an operationally feasible sampling time in the field. 

In this work, we present a methodology for BVOC collection as specified above. Our technique for BVOC collection was tested under field conditions for qualitative and semi-quantitative analyses of BVOCs emitted from *Q. ilex* plants at the atmospheric level. The study was carried out on two sampling sites: the first one was located in the experimental fields of CNR (National Research Council of Italy, Sesto Fiorentino, FI, Italy), where BVOCs were collected from three-year-old *Q. ilex* L. potted plants; the second experimental site consisted of a Mediterranean sclerophyll forest located in the Maremma Regional Reserve (Alberese, GR, Italy). The forest was dominated by *Q. ilex,* while other woody species were also present in a lower percentage, such as *Rubus* spp., *Phillirea latifolia, Cistus salviifolius, Quercus cerris, Pistacia lentiscus, Acer monspessulanum, A. campestre* and *Fraxinus ornus.*

Mediterranean forests are important for their ecological and socio-economic value providing several ecosystem services and goods to society [41,42,43]. To test our sampling method, we selected *Quercus ilex* as the most representative species of the Mediterranean forest ecosystem that, in recent years, has been subjected to repeated drought events [44]. Thus, the main aim of our study was to develop and test a new, rapid and simple sampling strategy to obtain reliable data on BVOC emission at the environmental level.

## 2. Results

### 2.1. BVOC Identification and Qualitative Analysis

Table 1 summarizes terpenes identified in the chromatograms (Figure S1) obtained from the analyses of samples collected at the three sampling points. In all three sampling points, the only terpenes identified were monoterpenes and monoterpenoids (MTs), while no sesquiterpenes or other BVOCs were detected. Indeed, the *Q. ilex* BVOC emission pattern is mainly characterized by monoterpenes [45,46,47,48,49,50]. In the first sampling point, there were no differences in MTs collected using the two sampling techniques (DBSS and static sampling). In both cases, the identified MTs were: monocyclic monoterpene hydrocarbons (d-limonene and *p*-cymene), one oxygenated derivative of monocyclic monoterpenes (1,8-cineole), and bicyclic monoterpene hydrocarbons (*α*-pinene, *α*-thujene, camphene, *β*-pinene, sabinene, car-3-ene). Similarly, qualitative differences between the two sampling techniques were not found in the second sampling point. In particular, the following acyclic monoterpene hydrocarbons were detected: myrcene and *β*-cis-ocimene. *β*-phellandrene, *α*-phellandrene, *α*-terpinene, d-limonene, *γ*-terpinene, *p*-cymene and terpinolene were detected among monocyclic monoterpene hydrocarbons. Finally, among the bicyclic monoterpene hydrocarbons, the following compounds were identified: *α*-pinene, *α*-thujene, camphene, *β*-pinene, sabinene and car-3-ene. Some qualitative differences were found during the third sampling point between DBSS and static sampling. Indeed, a higher number of MTs were identified using the DBSS compared to the static sampling. Among the acyclic monoterpene hydrocarbons, *β*-cis-ocimene were identified in both cases, while myrcene and *β*-trans-ocimene were found only using the DBSS. Among the monocyclic monoterpene hydrocarbons, d-limonene and *p*-cymene were detected using both sampling techniques, while only applying DBSS, *α*-terpinene, *γ*-terpinene and terpinolene were found. Finally, among the bicyclic monoterpene hydrocarbons, the following were found: *α*-pinene, *α*-thujene, camphene, *β*-pinene and sabinene in both cases, while car-3-ene only using DBSS.

### 2.2. Semi-Quantitative Analysis of Individual and Total MTs

**Figure 1:**
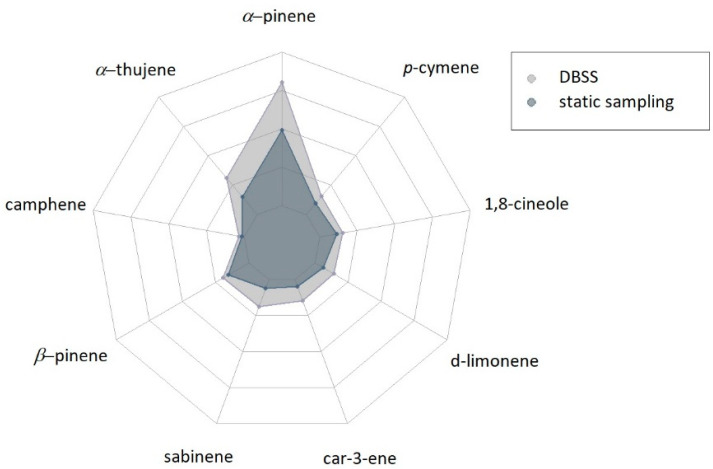
Radar-plot showing the results of the semi-quantitative analysis of BVOCs at sampling point 1 (*n* = 3) expressed as the peak area of each individual compound. The amounts of different compounds range between 0 and 350,000. Results of statistical analysis are reported in Table S1.

In the first sampling point (Figure 1), a similar trend was common for both sampling techniques. In particular, *α*-pinene resulted as the most abundant compound sampled using both techniques, while α-thujene was the second most abundant compound only in samples collected by the DBSS technique. All peak areas were higher in samples collected by DBSS compared to the static technique. 

**Figure 2:**
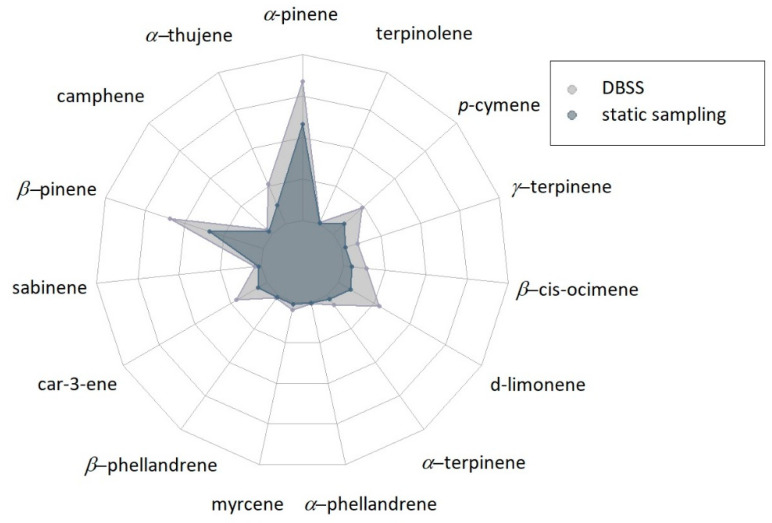
Radar-plot showing the results of the semi-quantitative analysis of BVOCs at sampling point 2 (*n* = 3) expressed as the peak area of each individual compound. The amounts of different compounds range between 0 and 2,000,000. Results of statistical analysis are reported in Table S2.

A similar trend was also found in the second sampling point (Figure 2). The most abundant compounds were *α*-pinene and *β*-pinene, followed by *α*-thujene, car-3-ene, d-limonene and *p*-cymene. Similar to sampling point 1, the amounts of the single compounds collected by DBSS were higher compared to the static technique.

**Figure 3:**
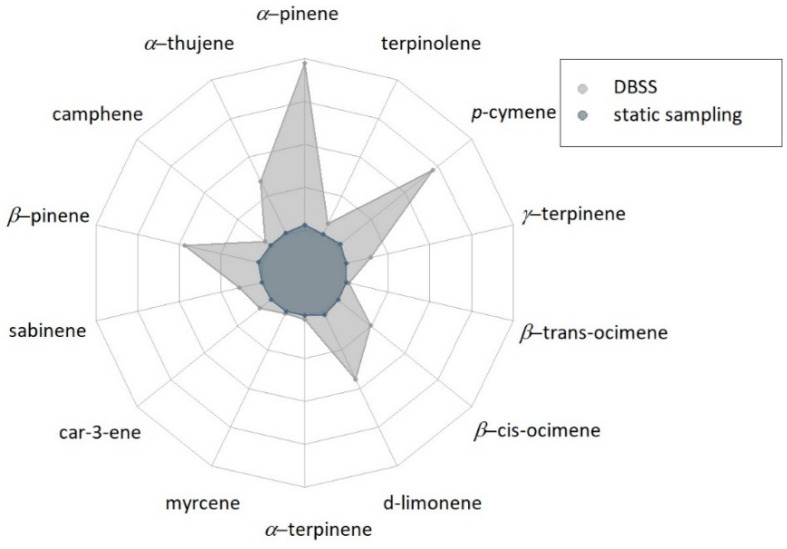
Radar-plot showing the results of the semi-quantitative analysis of BVOCs at sampling point 3 (*n* = 3) expressed as the peak area of each individual compound. The amounts of different compounds range between 0 and 1,350,000. Results of statistical analysis are reported in Table S3.

**Figure 4:**
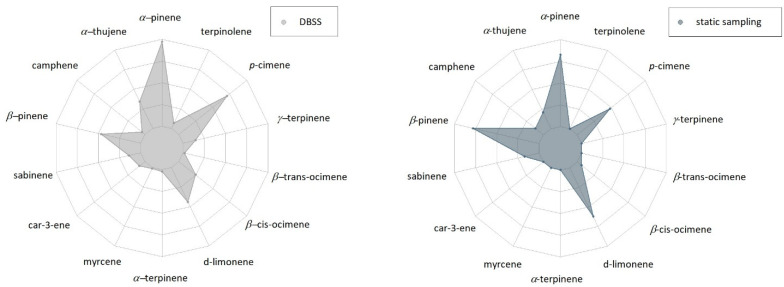
Radar-plots showing the results of the semi-quantitative analysis of BVOCs at sampling point 3 (*n* = 3) expressed as the peak area of each individual compound. The results are split in two graphs to highlight the quantitative differences between the two sampling techniques (different scales: DBSS from 0 to 1,350,000; static sampling from 0 to 45,000). Results of statistical analysis are reported in Table S3.

In the case of sampling point 3, the amounts of BVOCs collected using the DBSS were much higher than those found using the static sampling technique (Figure 3). In addition, at this sampling point, the DBSS allowed collecting a wider range of MTs compared to the samples collected using the static technique (Table 1). Indeed, compounds, such as car-3-ene, myrcene, *α*-terpinene, *β*-trans-ocimene, *γ*-terpinene and terpinolene, were detected only when using the DBSS. Finally, there is a slight difference in the trend of samples collected by DBSS and by static sampling. The samples collected by DBSS contained mainly *α*-pinene and *p*-cymene, followed by *β*-pinene and d-limonene, whereas in samples collected by the static technique, *α*-pinene and *β*-pinene were the most abundant compounds, followed by d-limonene and *p*-cymene (Figure 4).

**Figure 5:**
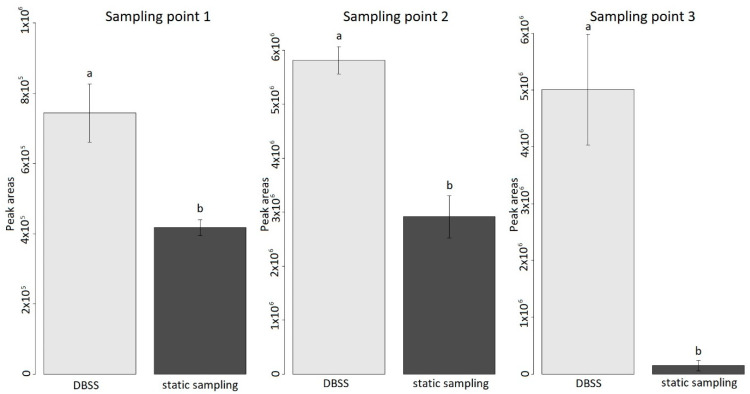
The amount of total MTs collected using DBSS (light grey) and static technique (dark grey) at sampling point 1, sampling point 2 and sampling point 3. Data are means ± standard deviation (*n* = 3). Data were analyzed by one-way ANOVA test, and the letters indicate statistical differences between the two sampling techniques in each sampling point obtained from a Tukey post hoc test (*p* ≤ 0.05). Results of statistical analysis are reported in Table S4.

Considering the total area of MTs in each sampling point, DBSS allowed collecting a higher total amount of MTs compared to the static technique (Figure 5). In particular, at sampling points 1 and 2, the sum of areas of MTs obtained from the DBSS was double compared to that of the static technique (56% for sampling point 1 and 50% for sampling point 2). In the third sampling point, the difference between the two techniques was considerably higher; indeed, the amounts of MTs collected by the static technique was only ~3% compared with the amounts obtained using the DBSS. 

## 3. Discussion

In our study, air samples collected by static and DBSS techniques were compared to evaluate the most efficient and sensitive methodology for BVOC collection under field conditions. It is important to notice that the sampling site at the Maremma National Reserve allowed us to test the DBSS technique in a complex environment, in which many plants that emit MTs were present. Indeed, in this sampling site, in addition to *Q. ilex*, other species such as *Rubus* spp., *Phillirea latifolia*, *Cistus salvifolius, Q. cerris*, *Pistacia lentiscus*, *Acer monspessulanum*, *A. campestre* and *Fraxinus ornus* were observed in a lower percentage. Finally, two additional sources of BVOCs in the forest could be soil microbes and litter. However, microbial BVOC emission rates are very low in Mediterranean shrublands [51]. In addition, as recently observed by Viros et al. [52], BVOCs emitted from *Q. ilex* litter do not include monoterpenes, thus representing a negligible source of these compounds. On this basis, we can suggest that the compounds found in our study derived from green leaves and not from litter or soil microbes.

We used DVB/CAR/PDMS coating type fiber, and the choice was dependent on its technical characteristics [30]. Additionally, our choice was dependent on the fact that, in previous studies, the extraction efficiency of DVB/CAR/PDMS coating for terpenoid was better than that of other fibers on the market [53,54]. Yassaa et al. [45] showed that DVB/CAR/PDMS fibers might have competitive adsorption of isoprenoids and saturation condition of the coating. Nonetheless, in our study, DVB/CAR/PDMS fibers were the most appropriate choice for sampling BVOCs in field conditions because the exposure of the fibers directly to the atmosphere did not allow them to reach the saturation of the fiber coating. Additionally, the use of an open, dynamic headspace system removed the problems related to static headspace, such as increases in temperature and humidity [31].

Sampling parameters, such as time window (11 am–3 pm), fan height (40–45 cm) and sampling duration (4 h), were chosen following previous studies (see below). Several authors [55,56,57] have shown that the higher emissions of BVOCs occur between 9 am and 5 pm, with a maximum peak around 1–2 pm [56]. Indeed, their emission is linked with the time when both temperatures and solar radiation were higher. The height of 45 cm from the ground was chosen since terpene concentrations have been demonstrated to be high at heights from the forest floor to 4 m. This height is optimal to limit wind action, as it allows for a more sheltered sampling condition [39]. Moreover, the choice of the height at which the fan sampler has been positioned was made to allow ease of operation during sampling (i.e., to expose and to retract the fiber; to turn on and off the fan). Lastly, the sampling duration was set to 4 h to have a long exposition time of SPME (to collect as many BVOCs as possible while in the pre-equilibrium case—[40]) and to allow a feasible sampling time in the field during every season. Indeed, longer acquisition times could be difficult to implement in winter and fall because unsuitable weather conditions are more frequent, such as strong winds and rain and shorter daylight availability. 

Our results showed that the DBSS was able to sample higher amounts of BVOCs in the two experimental sites (Sesto Fiorentino and Maremma National Reserve) at all sampling points (Figure 1, Figure 2, Figure 3, Figure 4 and Figure 5). Indeed, in the first and second sampling point, the total amount of MTs obtained using DBSS was double that of the static technique (Figure 5). The differences in the amount of BVOCs collected between the two techniques were particularly noticeable at sampling point 3 (Figure 5), in which qualitative differences were also observed (Figure 4). These qualitative differences observed at sampling point 3 may be explained by considering the lower air temperature recorded in October compared to June. Indeed, since BVOC emission is temperature-dependent, with an optimum around 25 °C, the collection of BVOCs using DBSS resulted particularly efficient when environmental conditions limited their emission [3,58]. Furthermore, a potential sink effect played by humidity, reducing BVOC adsorption in SPME fibers, could exacerbate this outcome since these compounds are water soluble at low concentrations [59]. Therefore, the collection of BVOCs without the fan-sampler system may reduce the BVOC adsorption on SPME fibers when present in traces or under windy weather conditions [60,61]. The observed variations in the scale of the total monoterpenes obtained between sampling point 1 and sampling points 2 and 3 (Figure 5) could be explained by the differences in plant characteristics and site conditions. Indeed, in sampling point 1, the studied plants were represented by 15 three-year-old *Q. ilex* potted plants, while in sampling points 2 and 3, BVOCs were collected in a natural forest with mature trees. Therefore, the dimensions of the canopy were very different, as well as the environmental conditions of the sampling. In addition, the potted *Q. ilex* plants, with a mean height of 1.2 m, were positioned at the center of an open field subjected to wind gusts that could have reduced the deposition of BVOCs into the fibers compared to the under-canopy conditions of the forest. Finally, the site of sampling point 1 was located in Sesto Fiorentino, a semi-urban area in which BVOC degradation by atmospheric oxidants could have been occurred [62]. In particular, since the sampling was conducted during the central hours of the day, eventual monoterpene oxidation would have been caused by the presence of hydroxy radicals (OH) [63]. However, these types of degradation products were not detected in our experiment.

It is important to mention that the range of total MTs obtained in sampling points 2–3, which were carried out in the same sampling area but in different seasons, provided similar results when employing the DBSS strategy. This finding shows the high potential and good repeatability of the DBSS strategy.

Another advantage of the DBSS could derive from the homogenization of the sample through the fan. The creation of turbulent airflow, obtained by combining the fan and the net in front of the SPME fiber. BVOCs under forest canopy can have heterogeneous concentrations [39], and the emissions are influenced by several factors: different seasons, meteorological conditions, sunlight exposure, altitude, tree species and damages from herbivores [3,8,64]. Altogether, our results demonstrated the potential and versatility of the DBSS for the rapid in situ measurement of BVOCs under different environmental conditions. In particular, the DBSS allowed identifying the typical compounds emitted by *Q. ilex*. Indeed, qualitative MTs identification carried out in our study is consistent with results reported in previous experiments conducted on the same species both in pots and in field conditions [49,50,65,66]. In all sampling points, *α*-pinene, *α*-thujene, *β*-pinene, sabinene and d-limonene were the most abundant compounds emitted by *Q. ilex* [5,46,48], which represented about 65–80% of the total detected monoterpenes. While other BVOCs such as car-3-ene, *p*-cymene and 1,8-cineole were also detected in lower amounts [46,48,67]. In addition, in agreement with Sabillo [47], our system was able to collect *β*-phellandrene, *α*-terpinene, *γ*-terpinene and terpinolene. Finally, consistently with Peñuelas et al. [58], we detected *α*-phellandrene (only at sampling point 2), emitted when *Q. ilex* is exposed to high air temperatures. 

The main compounds detected in our study were *α*-pinene and *α*-phellandrene, which have shown anti-inflammatory and anti-cancer properties, respectively [68,69,70]; moreover, it has been demonstrated that d-limonene and *p*-cymene can act against allergic lung inflammation [71,72], while *β*-pinene and 3-carene, have shown to possess anti-depressive and anxiolytic functions when inhaled [73]. Finally, monoterpenes, in general, and myrcene and 1,8-cineole, in particular, display neuroprotective roles thanks to their antioxidant effects [74,75]. For these reasons, it could be interesting to monitor the air quality and the emission of Mediterranean forest plants, to further investigate the healing effects of BVOCs on human health. 

## 4. Materials and Methods

### 4.1. Theoretical Background for SPME Sampling in Field Conditions

The SPME principle is explained by the Equation (1) [76]:

(1) $$n=c_{f}^{\infty }\;V_{f}=c_{0\;}\;\frac{K_{fs}\;V_{s}\;V_{f}}{K_{fs}V_{f}+V_{s}}$$

where *n* is the amount of analyte present in the sample matrix; *c*_0_ is the initial concentration of the analyte in the sample matrix; *C_f_^∞^* represents the concentrations in sample and fiber coating at equilibrium; *V_s_* and *V_f_* represent volumes of the sample and fiber coating, respectively, while *K_fs_* is the distribution coefficient of analyte between fiber coating and sample matrix. In this condition, whether the volume of sample (*V_s_*) is very large (e.g., in-field sampling), the equation becomes [76]:

(2) $$n=K_{fs}\;V_{f}\;c_{0}$$

Thus, Equation (2) indicates that, in the case of a large volume of sample, the extracted analyte amount will directly coincide with its concentration in the sample matrix, and it is not linked with the sample volume. Thus, for field sampling, the SPME fiber can be exposed directly to the specific environment, and under stable agitation conditions and constant temperature and extraction time, a quantitative analysis is possible at pre-equilibrium conditions [77,78].

### 4.2. Instrumental Setup

**Figure 6:**
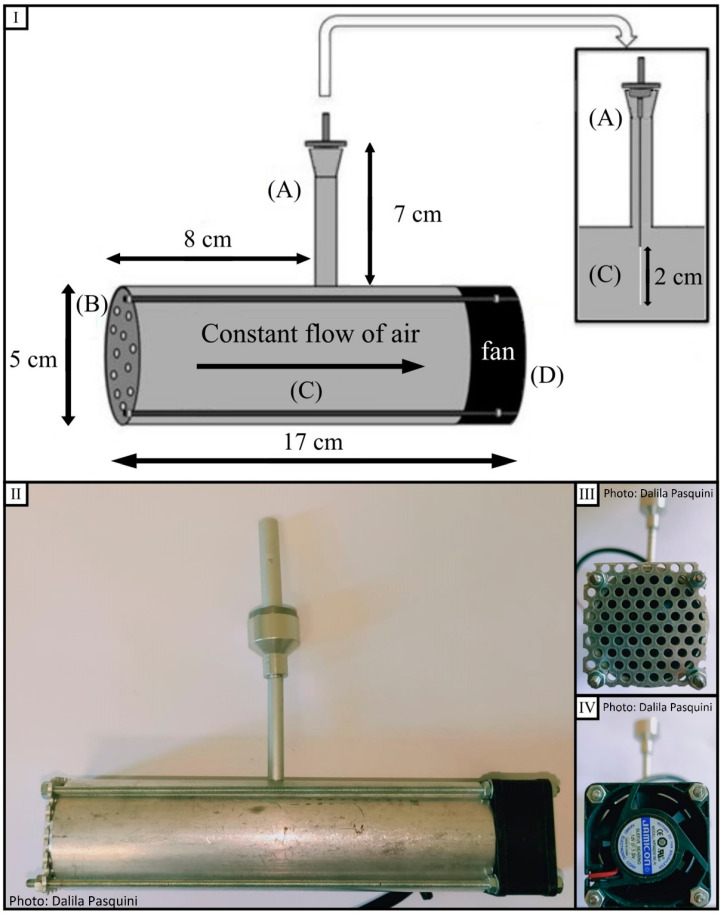
In **panel I**, the schematic drawing of the dynamic sampling system, adapted and modified by the BVOC system developed by Barreira et al. [36], is reported. The SPME fiber was firmly positioned in the appropriate vertical tube (**A**) by four small magnets. A metal net (**B**) was installed at one end of the cylinder (**C**) to protect the fiber, while at the opposite end, a fan (**D**) was positioned to create a swirling flow. All parts are linked together by screws. In **panel II**, a picture of the whole device is reported, while in **panel III** and **IV**, the details of the net located in the front of the device and the fan set on the back are shown.

The SPME fibers selected for the collection of BVOCs were divinylbenzene/carboxen/polydimethylsiloxane (DVB/CAR/PDMS-50/30 μm layer, Supelco, Sigma-Aldrich Co., Darmstadt, Germany). Each SPME fiber was held in the middle of a 17 cm long aluminum cylinder, perpendicularly connected to a smaller vertical cylinder 7 cm long acting as a support. In Figure 6, a blueprint (I) and pictures (II, III, IV) of the dynamic sampling system for BVOC collection are represented. Observing panel I on the top, the DBSS is constituted by a vertical tube (A), a metal net (B) connected to the main cylinder (C) both in aluminum, and a small fan (D). The diameter of the cylinder was chosen to allow a proper fiber exposition. One face of the cylinder (external diameter 5 cm and internal diameter 4 cm) is closed by a metal net (B) to protect the fiber from debris and to create a swirling flow, while the other face is closed by a small fan (Jamicon^®^, Kaimei Electronic Corp., New Taipei City, Taiwan, 12 V, 6200 rpm, 13 m^3^/h, 40 × 40 × 20 mm, panel IV) powered by a lead–acid battery (Join^®^, Alpha Elettronica S.r.l., Collecchio, Italy, 12 V, 4.5 AH). The instrument aluminum body is designed to allow an easier sampling and movement in the forest and is, therefore, light and resistant; in addition, to connect the single parts of the instrument, 4 long screws were used instead of glue to allow the utilization under high-temperature conditions and avoid any interferences during the sampling. 

After sampling, the fibers were placed in a special tray, within a hermetic case, with dedicated Teflon pressure supports to seal the needle until the fiber was transported to the laboratory, and all the fibers were subsequently desorbed within the same day, avoiding any degradation of compounds adsorbed into the SPMEs.

### 4.3. GC-MS Analysis

The desorption of SPME fibers was carried out with a 7820 A gas chromatograph coupled with a 5977E mass spectrometer (both from Agilent Technology, Santa Clara, CA, USA) operating in EI ionization mode at 70 eV energy. A DB-Wax (60 m × 250 μm × 0.5 μm, Agilent J&W) column was used for analytes separation. The other instrumental parameters were set as follows: injector temperature 260 °C, splitless mode and carrier flow (He) of 1.2 mL min^−1^. The oven temperature program was initially set at 40 °C for one minute, then increased by 5 °C/min until 210 °C, and then of 10 °C/min until 260 °C, at which temperature, it was held for 10 min, with a total run time of 48 min. The mass spectra were acquired in the 29–205 *m*/*z* range at three scans sec^−1^. Data were analyzed using the Agilent Mass Hunter software (Qualitative Analysis-Version B.06.00; Quantitative Analysis-Version B.07.01/Build 7.1.524.0), and the analytes were identified by matching their mass spectra and retention indices with those reported in NIST 11 spectral database library. Information from fragmentation patterns, retention times and data available from scientific literature was used for final identification [79,80]. The amounts of monoterpenes and monoterpenoids (MTs), expressed as peak areas, were reported both as single compounds and total MTs, and they have been related to Total Ion Current (TIC). 

### 4.4. Test of DBSS in Field Conditions

BVOC samplings were carried out in two different experimental sites, utilizing SPME fibers with both the static and dynamic sampling system (DBSS). The first sampling site was located in the experimental fields of CNR (National Research Council of Italy, 43°49′05″ N, 11°12′12″ E, Sesto Fiorentino, FI, Italy) on the 5th of June 2019. BVOCs emissions were collected from 15 3-year-old *Quercus ilex* L. potted plants (kindly supplied by Vivaio Matteini, 51100, Pistoia) maintained under optimal irrigation (sampling point 1). The heights of individual plants were about 120 cm with a stem diameter of 1 cm (Table 2). 

The second experimental site was located in the Maremma Regional Reserve at 320 m altitude (Alberese, 42°38′10″ N, 11°05′39″ E, Grosseto, GR, Italy), and BVOCs emissions were collected on the 20th of June 2019 (sampling point 2) and on the 1st of October 2019 (sampling point 3) (Table 2). This site was characterized by a Mediterranean sclerophyll forest with the predominance of *Q. ilex*, representing around 70% of the total number of individuals. At this site, SPME fibers were placed under the tree canopy, and the BVOC collection was carried out with the same settings mentioned above.

At both sampling sites, BVOC collection was conducted during rainless days and with light wind. The air temperature and humidity, as well as wind speed and direction, were recorded by weather stations installed in the proximity of the sampling sites by the Institute of Bioeconomy of the National Research Council of Italy (IBE, CNR). BVOCs were collected on DVB/CAR/PDMS coating fibers for 4 h, from 12 am to 4 pm, to cover the time interval with the highest presumable concentration of terpenes in the air as indicated in literature [60,61]. Indeed, several authors have shown a diurnal cycle in BVOCs emissions [55,56,57], with higher emissions between 9 am and 5 pm and a maximum peak around 1–2 pm [56]. The fibers were positioned at a height of 40–45 cm from the ground using a plastic band to connect them to a metal picket and at a distance of 30 cm from *Q. ilex* plants. All measurements were conducted in triplicate.

### 4.5. Statistical Analyses

All statistical analyses were carried out using R software (version 4.0.3). After carrying out the Shapiro and Levene tests, to check respectively the assumption of normality [81] and homoscedasticity [82,83], the data were analyzed using a one-way analysis of variance (ANOVA) and followed by a Tuckey post hoc test. 

## 5. Conclusions

In recent years, there has been an increasing interest in alternative, fast and easy-to-handle methods to sample BVOCs emitted in the field. This would be of particular importance for monitoring changes in terpene emissions by forests under both abiotic and biotic stresses, as well as for evaluating changes in air quality for human well-being. In order to develop an innovative sampling method to measure and analyze BVOCs under environmental conditions, the use of a DBSS technique could be a particularly efficient tool for a fast and simple BVOC collection in future fieldworks. This innovative sampling method is able to collect efficiently different classes of MTs at the environmental level, overcoming low-temperatures and high-humidity limitations typical of the static techniques and providing key information at the ecological level without the limitations of single plant measurements. Thus, a potential future application of this sampling device could be to monitor BVOCs’ environmental emissions from *Q. ilex* forests that experience high levels of tree mortality in comparison to healthy forests. Additional research, including comparison with other techniques (such as those employing cartridge sampling systems), acquisition of a larger dataset and development of a quantification procedure, are required in order to further improve the DBSS sampling strategy, which will provide quantitative data on BVOCs emitted at the environmental level. 

## Figures and Tables

**Table 1:** A list of the identified compounds by comparison with the NIST 11 library. Their names and retention times (RT) are presented. The peak number (n. Peak) corresponds to the numbers reported in the chromatograms (Figure S1). It is also reported the presence (y: yes) or the absence (n: no) of each compound in each the different sampling points (SP 1, SP 2 or SP 3) and in the two sampling conditions (dynamic/static).

n. Peak	Compound Identified	RT	Presence		
		(min)	SP 1 (dynamic/static)	SP 2 (dynamic/static)	SP 3 (dynamic/static)
1	*α*-pinene	10.07 ± 0.02	y/y	y/y	y/y
2	*α*-thujene	10.15 ± 0.02	y/y	y/y	y/y
3	camphene	11.35 ± 0.05	y/y	y/y	y/y
4	*β*-pinene	12.61 ± 0.04	y/y	y/y	y/y
5	sabinene	12.76 ± 0.05	y/y	y/y	y/y
6	car-3-ene	12.97 ± 0.03	y/y	y/y	y/n
7	*β*-phellandrene	13.10 ± 0.03	n/n	y/y	n/n
8	myrcene	14.12 ± 0.02	n/n	y/y	y/n
9	*α*-phellandrene	14.35 ± 0.02	n/n	y/y	n/n
10	*α*-terpinene	14.83 ± 0.04	n/n	y/y	y/n
11	d-limonene	15.39 ± 0.02	y/y	y/y	y/y
12	1,8-cineole	15.68 ± 0.01	y/y	n/n	n/n
13	*β*-cis-ocimene	15.72 ± 0.02	n/n	y/y	y/y
14	*β*-trans-ocimene	13.99 ± 0.02	n/n	n/n	y/n
15	*γ*-terpinene	16.80 ± 0.01	n/n	y/y	y/n
16	*p*-cymene	17.62 ± 0.02	y/y	y/y	y/y
17	terpinolene	17.95 ± 0.01	n/n	y/y	y/n

**Table 2:** The table shows the main atmospheric and sampling parameters: days of sampling, atmospheric temperature and humidity, speed and direction of wind, time window of sampling and site characteristics.

Sampling Point	Sampling Day	Temperature	Humidity	Wind	Sampling Time	Plants	Site
N	dd/mm/yyyy	°C	%	speed (km/h), direction			
1	6/6/2019	24 °C	45%	9–14 km/h, W/SW	12 pm 4pm	*Q. ilex* in pot	Sesto Fiorentino (FI)
2	20/06/2019	26.5 °C	45%	10 km/h, W/SW	12 pm 4 pm	*Q. ilex* forest	Maremma Regional Park (GR)
3	1/10/2019	20 °C	60%	8km/h, S	12 pm 4 pm	*Q. ilex* forest	Maremma Regional Park (GR)

## Data Availability

Data will be made available once the manuscript is accepted for the publication.

## Supplementary Materials

The following are available online at https://www.mdpi.com/article/10.3390/metabo11060388/s1, Figure S1: Chromatograms, Table S1: ANOVA summary sampling point 1, Table S2: ANOVA summary sampling point 2; Table S3: ANOVA summary sampling point 3; Table S4: ANOVA summary all sampling points (1,2 and 3).
