# Supplementary material for: An Improvement of SPME-Based Sampling Technique to Collect Volatile Organic Compounds from Quercus ilex at the Environmental Level
Source: Metabolites. 2021 Jun 14;11(6):388. doi: 10.3390/metabo11060388 (PMC8232123; doi:10.3390/metabo11060388)
Supplement: Supplementary file 1 [file metabolites-11-00388-s001.zip › Supp_material_revised/Supp_material_revised.pdf]

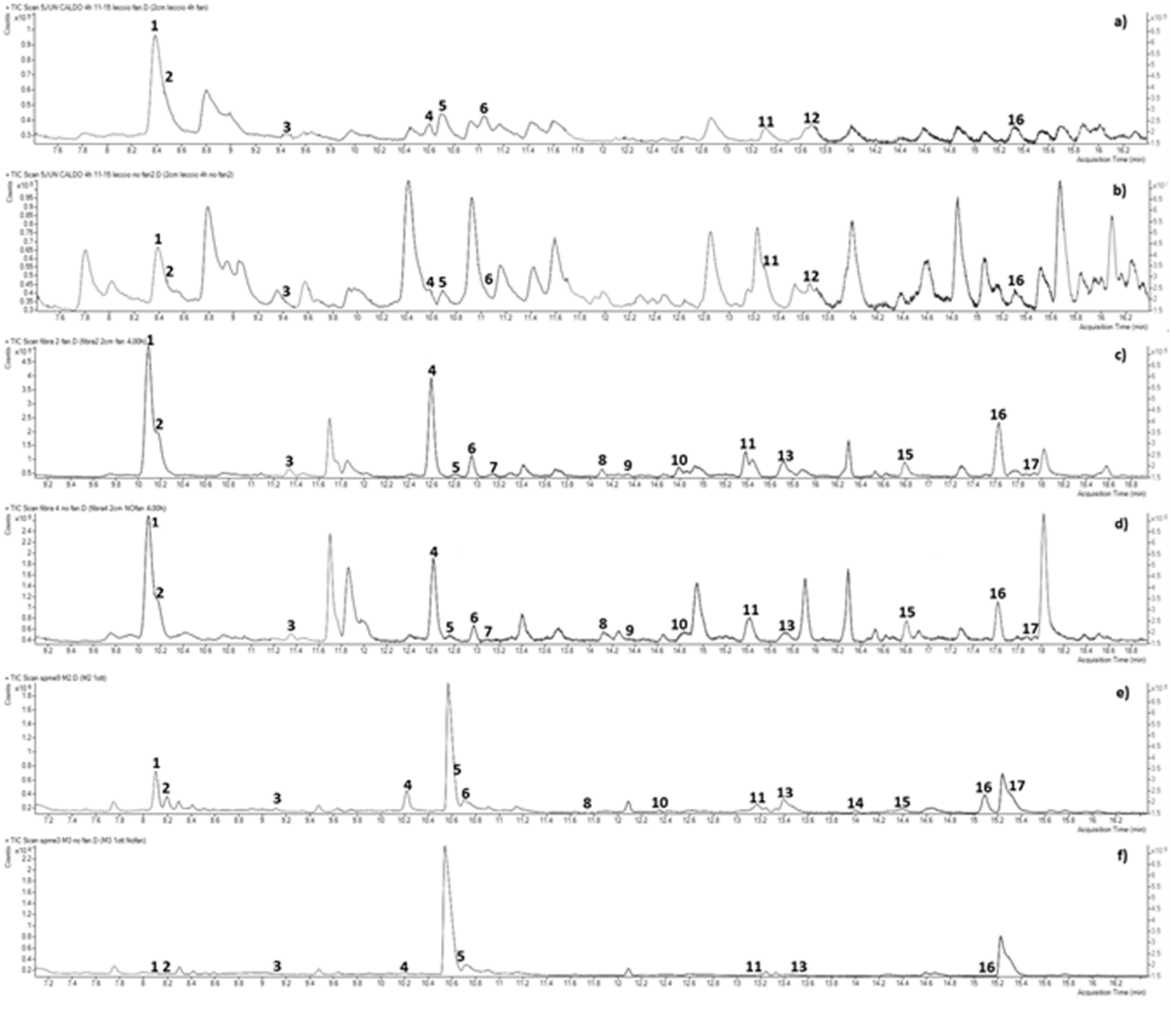

Figure S1. Representative Total Ion Chromatograms (TIC) for each sampling point using the two different methods (DBSS and static sampling): a) DBSS at sampling point 1; b) static method at sampling point 1; c) DBSS at sampling point 2; d) static method at sampling point 2; e) DBSS at sampling point 3; f) static method at sampling point 3. The numbered peaks correspond to: (1) α-pinene, (2) α-thujene, (3) camphene, (4) β-pinene, (5) sabinene, (6) car-3-ene, (7) β-phellandrene, (8) myrcene, (9) α-phellandrene, (10) α-terpinene, (11) d-limonene, (12) 1,8-cineole, (13) β-cis-ocimene, (14) β-trans-ocimene, (15) γ-terpinene, (16) *p*-cymene, (17) terpinolene. The shift in the retention time observed in the peaks in the chromatograms obtained in sampling point 3 (e-f) is due to a difference in the length of the column.

Table S1. Statistical results obtained from one-way Analysis of Variance (ANOVA) of the amounts of compounds found in sampling point 1, using DBSS and static sampling techniques. (ns p>0.05; * p<0.05; ** p<0.01; ***p<0.001).

**Compounds**	**F value**	***p***	
α-pinene	24.14	0.0080	**
α-thujene	10.65	0.0310	*
camphene	23.04	0.0086	**
β-pinene	0.12	0.7430	ns
sabinene	3.15	0.151	ns
car-3-ene	67.59	0.0012	**
d-limonene	7.66	0.0505	ns
1,8-cineole	4.81	0.0934	ns
p-cymene	1.84	0.2460	ns

Table S2. Statistical results obtained from one-way Analysis of Variance (ANOVA) of the amounts of compounds found in sampling point 2, using DBSS and static sampling techniques. (ns p>0.05; * p<0.05; ** p<0.01; ***p<0.001).

**Compounds**	**F value**	***p***	
α-pinene	5.27	0.0833	ns
α-thujene	38.78	0.0034	**
camphene	33.54	0.0044	**
β-pinene	25.16	0.0074	**
sabinene	33.3	0.0045	**
car-3-ene	3.46	0.1370	ns
β-phellandrene	3.97	0.1170	ns
myrcene	12.30	0.0247	*
α-phellandrene	47.88	0.0023	**
α-terpinene	54.69	0.0018	**
d-limonene	69.81	0.0011	**
β-cis-ocimene	119.40	0.0004	***
γ-terpinene	46.55	0.0024	**
p-cymene	9.81	0.0351	*
terpinolene	11.20	0.0286	*

Table S3. Statistical results obtained from one-way Analysis of Variance (ANOVA) of the amounts of compounds found in sampling point 3, using DBSS and static sampling techniques. (ns p>0.05; * p<0.05; ** p<0.01; ***p<0.001)

Compounds	F value	*p*	
α-pinene	30.56	0.0052	**
α-thujene	48.34	0.0022	**
camphene	86.29	0.0007	***
β-pinene	28.77	0.0058	**
sabinene	27.92	0.0061	**
car-3-ene	17.74	0.0136	*
myrcene	33.69	0.0044	**
α-terpinene	17.50	0.0139	*
d-limonene	101	0.0005	***
β-cis-ocimene	19.39	0.0117	*
β-trans-ocimene	43.63	0.0027	**
γ-terpinene	36.16	0.0038	**
p-cymene	55.03	0.0018	**
terpinolene	34.28	0.0042	**

Table S4. Statistical results obtained from one-way Analysis of Variance (ANOVA) of the total amounts of compounds found in each sampling points (1, 2, 3), using DBSS and static sampling techniques. (ns p>0.05; * p<0.05; ** p<0.01; ***p<0.001)

Sampling point	F value	*p*	
1	43.77	0.0027	**
2	114.1	0.0004	***
3	73.63	0.0010	**
