# Supplementary figures and images for: An Improvement of SPME-Based Sampling Technique to Collect Volatile Organic Compounds from Quercus ilex at the Environmental Level
Source: Metabolites. 2021 Jun 14;11(6):388. doi: 10.3390/metabo11060388 (PMC8232123; doi:10.3390/metabo11060388)

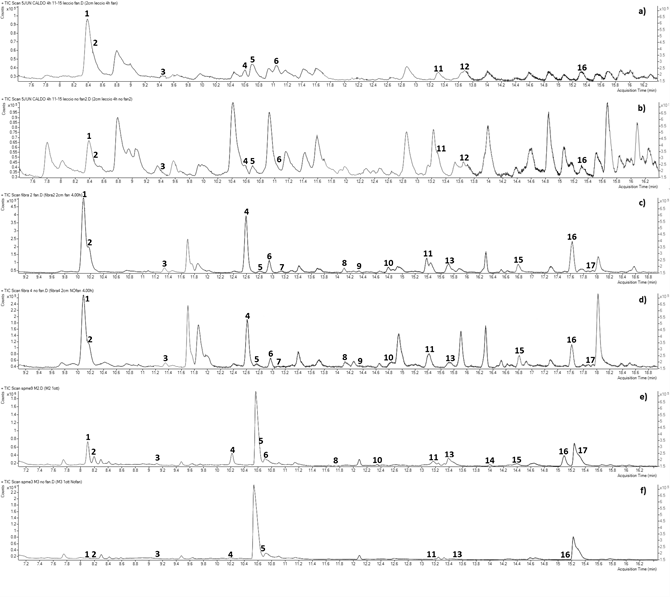

Supplement: Supplementary file 1 [file metabolites-11-00388-s001.zip › Supp_material_revised/FigureS1.png]
